# Effectiveness and Safety of Electroacupuncture on Poststroke Urinary Incontinence: Study Protocol of a Pilot Multicentered, Randomized, Parallel, Sham-Controlled Trial

**DOI:** 10.1155/2016/5709295

**Published:** 2016-11-30

**Authors:** Seungwon Shin, Jiwon Lee, Ami Yu, Junghee Yoo, Euiju Lee

**Affiliations:** ^1^Department of Clinical Korean Medicine, Graduate School, Kyung Hee University, 26 Kyungheedae-ro, Dongdaemun-gu, Seoul 02447, Republic of Korea; ^2^Korean Medicine Clinical Trial Center, Kyung Hee University Korean Medicine Hospital, 23 Kyungheedae-ro, Dongdaemun-gu, Seoul 02447, Republic of Korea; ^3^Stroke Center, Kyung Hee University Korean Medicine Hospital, 23 Kyungheedae-ro, Dongdaemun-gu, Seoul 02447, Republic of Korea; ^4^College of Nursing Science, Kyung Hee University, 23 Kyungheedae-ro, Dongdaemun-gu, Seoul 02447, Republic of Korea; ^5^College of Korean Medicine, Kyung Hee University, 23 Kyungheedae-ro, Dongdaemun-gu, Seoul 02447, Republic of Korea

## Abstract

This pilot multicentered, randomized, parallel, sham-controlled trial is intended to evaluate the effectiveness and safety of electroacupuncture therapy for poststroke patients with urinary incontinence. Forty stroke survivors aged >19 years will be recruited in 2 hospitals in the Republic of Korea. Patients who experienced stroke within 2 years and satisfy criteria of urinary frequencies ≥2 with either 3 to 4 points on the Patient Perception of Intensity of Urgency Scale or 13 points or more on the Korean version of the International Prostate Symptom Scale (K-IPSS) will be identified, along with other eligibility criteria. Patients will be randomly allocated to either a treatment or control group to receive 10 sessions of electroacupuncture or sham therapies, respectively. Patients and outcome assessors will be blinded. The primary outcome is the change of Total Urgency and Frequency Score between the baseline and the trial endpoint. The K-IPSS, the International Consultation on Incontinence Questionnaire for Urinary Incontinence Short Form, and the Lower Urinary Tract Symptoms Outcome Score will be evaluated for effectiveness assessment. Adverse events will be reported after every session. The Blinding Index will also be calculated. Data will be statistically analyzed with 0.05 significance levels by 2-sided testing.

## 1. Background

Urinary incontinence (UI) is defined as involuntary loss of urine [1], with reduced ability either to be aware of bladder signals before leakage or to take notice of eventual leakage or both [2]. The damaged neurological lesions in the brain of stroke survivors lead to loss of control of detrusor function, which is the reason that poststroke patients often experience UI [3, 4].

A previous study reviewed 9 papers from European countries, USA, and Japan and reported that the UI prevalence in stroke patients was estimated by 32% to 79% at admission and 25% to 28% at discharge [4]. A study suggested that the prevalence of poststroke urinary incontinence (PSUI) might be as high as 45.6% in Korea, which is higher than that of UI [5].

Urinary dysfunction generally affects the quality of life in stroke survivors [6], but the development of UI following a stroke is also a predictor of future functional recovery after acute stroke [7], with a strong association with both increased mortality rates and poor functional outcomes [8]. As a result, clinicians and researchers have been highly interested in the management or treatment of poststroke UI.

Recommended treatments for poststroke UI include bladder retraining, timed or prompted voiding, intermittent catheterization, the use of anticholinergic medications, and environmental and lifestyle support, none of which have sufficient evidence of efficacy, according to a systematic review [8]. Acupuncture therapies have been also explored for the management of UI after stroke in China. For example, stroke survivors who received acupuncture showed lower rates of UI than those who received no treatment or usual care, even though the quality of studies was questionable [8]. Another review implied that acupuncture or acupressure therapies exhibited favorable effects on UI patients with overactive bladder symptoms and suggested that a more qualified randomized controlled trial (RCT) should be implemented [10]. A Chinese study conducted a multicenter RCT and reported the positive clinical efficacy of electroacupuncture (EA) for apoplectic UI compared with indwelling catheterization [11].

However, there have been no RCTs to determine whether EA is effective for UI in poststroke patients compared with a sham comparator. This pilot study is aimed at evaluating the effectiveness and safety of EA therapy for poststroke patients with UI, to explore suitable conditions for a future full-scale RCT.

## 2. Methods

### 2.1. Study Overview 

#### 2.1.1. Design and Setting

This is a pilot multicentered, randomized, parallel, sham-controlled trial. Two hospitals in Korea (Kyunghee University Korean Medicine Hospital in Seoul and Wonkwang University Gwangju Medical Center in Gwangju) will recruit the participants. The Consolidated Standards of Reporting Trials (CONSORT) [12], Standards for Reporting Interventions in Clinical Trials of Acupuncture (STRICTA) [13], and Standard Protocol Items: Recommendations for Interventional Trials (SPIRIT) 2013 [9] guidelines were followed for development of this trial protocol. A schematic diagram to summarize the schedule of enrollment, interventions, assessments, and visits for participants, recommended by SPIRIT 2013, is depicted in Table 1.

#### 2.1.2. Ethical Committee Approval

The institutional review board in Kyung Hee University Korean Medicine Hospital approved this trial (KOMCIRB-160215-HR-006). The protocol was registered at ClinicalTrials.gov on Jun 29, 2016 (identifier no. NCT02819336).

### 2.2. Study Participants

#### 2.2.1. Inclusion Criteria

Forty patients diagnosed with PSUI will be recruited, satisfying all the following inclusion criteria: (a) male or female aged over 19 years; (b) survivor of stroke due to cerebral hemorrhage or infarction diagnosed by computed tomography or magnetic resonance imaging within 2 years; (c) patient with 2 or more urinary frequencies with 3 to 4 points on the Patient Perception of Intensity of Urgency Scale (PPIUS) or who scored 13 points or more on the Korean version of the International Prostate Symptom Scale (K-IPSS); and (d) any volunteer who signed an informed consent form.

#### 2.2.2. Exclusion Criteria

We will screen out patients who meet any of the following exclusion criteria: (a) 1 or more postvoiding residual volumes >200 mL per day; (b) clinically significant stress UI diagnosed on cough induction test or investigator's clinical experience; (c) recurrent urinary tract infection (UTI), defined as 4 or more UTIs requiring treatment in the prior 1 year; (d) cognitive impairment with 23 points or less on the Korean version of the Mini-Mental State Examination; (e) acute or chronic lower UTI examined by urine culture; (f) preexisting UI before the stroke occurred, with UI symptoms since then; (g) any major diseases in the lower urinary tract; (h) coagulation disorders; (i) medical or surgical procedures for peripheral arterial diseases; (j) psychiatric diseases; (k) fear of acupuncture; (l) pregnancy (examined by urine test); or (m) any other appropriate reason based on the clinical experience of the primary or subinvestigator. The investigators will take and record the medical history to determine the existence of criteria (f), (g), (h), (i), and (j).

Screening assessment will be conducted after we obtain informed consent forms from all voluntary candidates. For patients aged 75 years or older, we will offer more easily understood literature and consent forms. A copy of the signed consent will be given to the participants.

#### 2.2.3. Dropout Criteria

Participants will be dropped out when any one of the following criteria apply: (a) any of the aforementioned exclusion criteria is found after the patient screens in; (b) patients or their legal representatives withdraw consent or want to suspend participation; (c) investigators lose contact with the participant; (d) investigators or patients violate the trial protocol; (e) patients have serious or permanent injury from the trial; (f) patients take any drugs that can affect the results of the trial without direction by the investigators during the trial; or (g) patients have other sufficient reasons to interrupt the trial progress, as determined by investigators' clinical experience.

#### 2.2.4. Sample Size Calculation

Because there is no preceding study to evaluate the effectiveness of EA for PSUI using the Total Urgency and Frequency Score (TUFS), with a control of any sham devices, we reviewed a statistical study on the appropriate group size for a pilot study. One study suggested that at least 12 participants should be recruited in each group to conduct a pilot trial [14], and another suggested that an increase in size from 12 to 18 or 24 narrowed the confidence interval [15]. Accordingly, we decided that 18 participants per group would be adequate. Therefore, with anticipated dropout rates of 10%, totally 40 participants (20 per group) will be randomized and allocated each EA or sham acupuncture (SA) group.

### 2.3. Randomization and Group Allocation

An independent statistician will generate a singular sequence of random numbers using the R program (R Foundation for Statistical Computing, Vienna, Austria. http://www.r-project.org/) for the entire study. The participants will be given a unique screening number by the order of signing of informed consent, and they will be randomly allocated to either the EA or SA group.

After requesting group allocation, the investigators will receive an email or text message from the statistician including the group assignments for the patients. The allocation ratio is 1 : 1 (20 in the EA group or SA group, resp.).

The electronic file with random sequence and allocation information can be accessed by the independent statistician until the trial cessation or closeout.

### 2.4. Blinding

Patients and outcome assessors will be blinded throughout the trial. Patients will not be notified until the end of the trial about their group assignment. In addition, using the same guide tubes and turning on the EA apparatus in both groups will help keep the patients blinded during the study. The blinding assessment will be performed at the end of the trial using a Blinding Index (BI) [16]. Patient outcomes will be assessed by an independent study staff, who will assist in filling in the patient-rated questionnaires. Only when serious adverse events are reported can the group allocation be uncovered.

### 2.5. Interventions and Procedures

#### 2.5.1. Treatment Group

The intervention group will receive EA therapy using the following procedures. (a) The patient will be placed in lateral decubitus position. (b) The practitioners will install the guide tubes from the Park sham device at CV2, CV3, CV4, CV6, and bilateral points of SP6 and SP11 (8 acupoints in total). The location of acupoints conforms to World Health Organization guidelines [17]. (c) The verum acupuncture needles (stainless steel, 0.25 mm in diameter and 4.0 mm in length, Dong Bang Acupuncture Inc., Republic of Korea) are inserted at each acupoint through the guide tube to a depth of 10–15 mm. (d) After de qi response is elicited, electrical stimulation is applied for 20 minutes at middle frequency (30 Hz) (STN-111, Stratek, Republic of Korea).

#### 2.5.2. Sham Group

The control group will receive Park sham therapy using the following procedures. (a) The patient will be placed in lateral decubitus position. (b) The practitioners will install the same guide tubes at the same acupoints as in the EA group. (c) Nonpenetrating SA is implemented at each acupoint through the guide tubes. (d) Electrical stimulation is applied for 20 minutes at middle frequency (30 Hz), even though the stimulation is not delivered because the needles have not penetrated the skin.

The treatment session for both groups will be repeated 10 times for 18 days (the visit window is ±3 days). The patients will not take more than one session per day. Acupuncture practitioners with at least 1 year of clinical experience will be involved in the procedures. The practitioners will only explain how many needles will be used, where they are going to place the needles, and how long the needles will remain in place during the session.

The acupoints were selected based on a literature review of Korean acupuncture theories and expert consensus reached after discussion. The selected acupoints are usually used for bladder and genital disorders, including the general symptom of residual urine [18].

#### 2.5.3. Concomitant Medications

According to clinical practice guidelines [19], conventional treatments for stroke and complex management with drugs (antiplatelet agents, anticoagulants, or neuroprotectants), rehabilitation therapies, traditional herbal medications, EA (except for the CV2, CV3, CV4, CV6, SP6, and SP11 acupoints), acupuncture, and moxibustion will be allowed during the study for both groups. In addition, conventional treatments for UI such as oral medications, including diuretics, bladder rehabilitation training, traditional herbal medications, EA (except for the CV2, CV3, CV4, CV6, SP6, and SP11 acupoints), acupuncture, and moxibustion are permitted. However, treatment strategies of fluid therapy and oral administration of any UI drugs should not be changed from 3 days before the trial to the end, because they can significantly affect the symptoms of UI.

### 2.6. Outcome Assessment

One primary outcome (TUFS) and 3 secondary outcomes (International Consultation on Incontinence Questionnaire for Urinary Incontinence Short Form; ICIQ-UI-SF, K-IPSS, and Lower Urinary Tract Symptoms Outcome Score) will be evaluated for effective assessment.

#### 2.6.1. Primary Outcome

The change in TUFS will be primarily evaluated. PPIUS is a self-rated diary reporting time points, frequency of urination, and the intensity of urine urgency in 24 hours. The patient's perception of intensity is scored from 0 (no urgency) to 4 (urgency incontinence). TUFS is calculated using the sum of the PPIUS scores. This scale has been validated to assess the severity of overactive bladder and Lower Urinary Tract Symptoms, combining the symptoms of urgent and frequent urination [20]. The patient will be asked to report the scale for 24 hours at baseline and at the end of the trial, respectively.

#### 2.6.2. Secondary Outcomes

The change in quality of life for the PSUI between baseline and study closeout will be secondarily evaluated using ICIQ-UI-SF. The questionnaire consists of 3 items, asking (a) the frequencies of UI, (b) the amount of UI, and (c) how strongly the UI affects daily life. The total score will be 0 to 21. The validity and reliability of the scale were analyzed in a preceding study [21].

In the K-IPSS, general symptoms related to the lower urinary tract will also be assessed. The total score (0–35) is calculated for 7 items of 0–5 points each, and the severity will be considered mild (0–7 points), moderate (8–19 points), or severe (20–35 points). The scale includes questions related to prostate symptoms and quality of life [22, 23] and the Korean version of the scale was validated separately [24]. The change in score between the baseline and the study endpoint will be calculated and analyzed.

Finally, the Lower Urinary Tract Symptoms (LUTS) Outcome Score will also be included. This scale can comprehensively evaluate the objective and subjective severity of symptoms. Using the form validated in 2005 [25], we will evaluate the difference in scores before and after the trial.

#### 2.6.3. Safety Assessment

Outcome assessors will investigate any adverse events for safety of EA intervention in PSUI after every treatment session. Investigators will make a decision about the severity (mild, moderate, or severe), seriousness, and causality (definitely related, probably related, possibly related, possibly not related, definitely not related to the intervention, or not assessable) of the reported adverse events.

#### 2.6.4. Blinding Assessment

As described above, the patients will be asked which group they think they belong to after the final session to calculate the BI [16].

### 2.7. Statistical Analysis

Categorical variables will be presented with frequencies and percentage and analyzed by *χ* ^2^ test or Fisher's exact test for demographic information and adverse events. Continuous variables will be presented with mean ± standard deviation and analyzed by two-sample *t*-test or Mann-Whitney *U*-test for demographic information and effectiveness outcomes, depending on the normality of the baseline distribution. If necessary, efficacy data will be evaluated with analysis of covariance (ANCOVA) with covariates of recruiting center and any demographic information, such as gender.

Full analysis set (FAS) (defined as the data set of the participants who receive 7 or more EA or SA sessions and provide the primary outcome at least once) will be primarily used for the effectiveness assessment. Per-protocol (PP) analysis (defined as the data set of the participants who complete every 10 EA or SA session) will be subordinately adopted. Missing data will be imputed by the last observation carried forward method. For the safety assessment, safety set analysis will be performed with the data obtained from all of the participants who receive at least one EA or SA session.

Finally, for the BI assessment, the distribution of actual allocation and participant's responses (EA group, SA group, or DO NOT KNOW) will be presented with 2 × 3 cross tabulation along with the index calculation.

IBM SPSS Statistics for Windows (Version 21.0. IBM Corp. Released 2012. Armonk, NY, USA) will be used, with 0.05 significance levels by a 2-sided test.

## 3. Discussion

This study protocol is intended to conduct a pilot multicentered, randomized, parallel, sham-controlled trial, evaluating the effectiveness and safety of EA, compared with SA, for PSUI patients. The primary effectiveness will be measured by a patient-reported scale, TUFS, with other outcomes, namely, ICIQ-UI-SF, K-IPSS, and LUTS Outcome Score. The safety will be analyzed with adverse events reporting.

According to a review study on acupuncture for voiding disorders after stroke, CV4, SP6, CV3, and CV6 are the 4 acupoints that have been most used in 16 clinical studies [26]. These acupoints are known to control the kidney or body fluid and can possibly manage bladder problems, according to the literature on Korean traditional medicine [18].

Park sham is a placebo acupuncture that was developed to make patients indistinguishable from those who receive genuine needling [27]. With use of a specially developed guide tube, patients cannot observe the needle penetrating through the skin. We will use this guide tube to blind participants, even in the actual EA group. The validation test for SA has already been performed [28].

We designed this trial as a pilot study because no preceding study exists. With the results, we expect to explore more adequate trial settings and calculate statistically powered sample sizes for a full-scale clinical trial to confirm the effectiveness and safety of EA therapies for UI in stroke survivors.

## Figures and Tables

**Table 1:** Study schedule with enrollment, interventions, and assessment plan.

	Study period												
	Enrollment	Allocation	Postallocation										Closeout
Visits	V1	V2	V3	V4	V5	V6	V7	V8	V9	V10	V11	V12	V13

Time point	Day (−1)	Day 0	Days 1–18 (±3 days)										Day 18 (±3 days)

Enrollment													
Informed consent	X												
*[Demographic information]*	X												
*[Medical history]*	X												
*[Concomitant medication]*	X												
*[Laboratory test* ^*∗*^ *]*	X												
*[Uroflowmetry & bladder scan]*	X												
*[PPIUS]*	X												
*[K-IPSS]*	X												
*[MMSE-K]*	X												
Eligibility screen	X												
Random allocation		X											

Interventions													
*[Treatment: electroacupuncture]*			⧫	⧫	⧫	⧫	⧫	⧫	⧫	⧫	⧫	⧫	
*[Control: sham acupuncture]*			⧫	⧫	⧫	⧫	⧫	⧫	⧫	⧫	⧫	⧫	

Assessments													
*[PPIUS/TUFS]*		X											X
*[ICIQ-UI-SF]*		X											X
*[K-IPSS]*		X											X
*[LOS]*		X											X
*[Blinding Index]*													X
*[Adverse events]*			X	X	X	X	X	X	X	X	X	X	

This table has been modified from the example template recommended by SPIRIT guideline [9].

^*∗*^Blood test: PSA (prostate specific antigen) test for male participants. Urine test: urinalysis and urine culture for all participants, HCG (human chorionic gonadotropin) test for fertile female participants

MMSE-K: the Korean version of Mini-Mental State Examination; PPIUS: Patient Perception of Intensity of Urgency Scale; TUFS: Total Urgency and Frequency Score; ICIQ-UI-SF: International Consultation on Incontinence Questionnaire for Urinary Incontinence Short Form; K-IPSS: The Korean version of International Prostate Symptom Scale; LOS: Lower urinary track system Outcome Score.
