# Corrigendum to “Effectiveness and Safety of Electroacupuncture on Poststroke Urinary Incontinence: Study Protocol of a Pilot Multicentered, Randomized, Parallel, Sham-Controlled Trial”

**DOI:** 10.1155/2017/1720793

**Published:** 2017-04-05

**Authors:** Seungwon Shin, Jiwon Lee, Ami Yu, Junghee Yoo, Euiju Lee

**Affiliations:** ^1^Department of Clinical Korean Medicine, Graduate School, Kyung Hee University, 26 Kyungheedae-ro, Dongdaemun-gu, Seoul 02447, Republic of Korea; ^2^Stroke Center, Kyung Hee University Korean Medicine Hospital, 23 Kyungheedae-ro, Dongdaemun-gu, Seoul 02447, Republic of Korea; ^3^Korean Medicine Clinical Trial Center, Kyung Hee University Korean Medicine Hospital, 23 Kyungheedae-ro, Dongdaemun-gu, Seoul 02447, Republic of Korea; ^4^College of Nursing Science, Kyung Hee University, 23 Kyungheedae-ro, Dongdaemun-gu, Seoul 02447, Republic of Korea; ^5^College of Korean Medicine, Kyung Hee University, 23 Kyungheedae-ro, Dongdaemun-gu, Seoul 02447, Republic of Korea

In the article titled “Effectiveness and Safety of Electroacupuncture on Poststroke Urinary Incontinence: Study Protocol of a Pilot Multicentered, Randomized, Parallel, Sham-Controlled Trial” [1], the first author's affiliation should be changed to “Department of Clinical Korean Medicine, Graduate School, Kyung Hee University, 26 Kyungheedae-ro, Dongdaemun-gu, Seoul 02447, Republic of Korea” only. The corrected author and affiliations lists are shown above.
